# Supercritical CO_2_ Extraction of *Eruca sativa* Using Cosolvents: Phytochemical Composition by LC-MS Analysis

**DOI:** 10.3390/molecules23123240

**Published:** 2018-12-07

**Authors:** Stefania Sut, Irene Boschiero, Miriam Solana, Mario Malagoli, Alberto Bertucco, Stefano Dall’Acqua

**Affiliations:** 1 DAFNAE, Department of Agronomy, Food, Natural Resources, Animals and Environment, Agripolis Campus, University of Padova, 35020 Legnaro (PD), Italy; stefania.sut@studenti.unipd.it (S.S.); mario.malagoli@unipd.it (M.M.); 2 Department of Pharmaceutical and Pharmacological Sciences, University of Padua, Via Marzolo 5, 35131 Padua, Italy; boschieroirene@gmail.com; 3 Department of Industrial Engineering DII, University of Padova, Via Marzolo 9, 35131 Padua, Italy; mirivillas@gmail.com (M.S.); alberto.bertucco@unipd.it (A.B.)

**Keywords:** *Eruca sativa*, supercritical CO_2_, glucosinolate, antioxidant activity

## Abstract

Background: *Eruca sativa* Mill. is a good source of glucosinolates (GLS), phenolic compounds and unsaturated fatty acids, being a valuable material for the production of functional-foods or nutraceutical ingredients. Extraction by supercritical CO_2_ (SCO_2_) can be used and the limitations due to the apolar nature of CO_2_ can be overcome using co-solvents. In this paper different cosolvents and conditions were used for SCO_2_ extraction and the composition of the obtained extracts was studied by LC-MS. Results: Water resulted the ideal co-solvent, allowing the extraction of glucosinolates in comparable amounts to the classical procedure with boiling water, as it can be carried out at mild temperatures (45 °C vs. >100 °C). Increasing the pressure improved the GLS extraction. On the other hand polyphenol extraction under the studied conditions was not influenced by pressure and temperature variations. The in vitro antioxidant effect of the obtained extracts was also measured, showing significant activity in the DPPH and FC tests. Conclusions: The GLS, flavonoids and lipids composition of the obtained extracts was studied, showing the presence of numerous antioxidant constituents useful for nutraceutical applications. The extraction method using SCO_2_ and water as co-solvent presents advantages in terms of safety because these solvents are generally recognised as safe. Water as cosolvent at 8% resulted useful for the extraction of both glucosinolates and phenolics in good amount and is environmentally acceptable as well as safe for food production.

## 1. Introduction

Many cruciferous plants contain a number of nutrients and phytochemicals that are associated with health promoting effects, in particular glucosinolates, that are responsible for their typical pungent aromas and spicy taste [1,2]. Such compounds are considered health promoting constituents and have been examined as such in different in vitro, in vivo, clinical and epidemiological studies [3,4,5]. *Eruca sativa* (Brassicaceae) known as “rocket salad” is very well known vegetable that is widely consumed in many countries, being popular in mixed salads due to its distinctive taste and textural appearance [1,6]. Rocket contains a large number of bioactive constituents, and especially due to its content in glucosinolates, phenolics and unsaturated fatty acids, can be considered a good source of health promoting compounds [1,6,7,8,9,10] Rocket salad is not only a healthy food, but could be useful as a valuable source for the preparation of extracts to be used in health-promoting products. In recent years there has been a growing interest of consumers towards these products, especially in the form of food supplements, nutraceuticals or functional-foods [7,11,12,13]. Such products are not medicinal, are aimed to maintain or promote health and are frequently obtained using plant extracts. Due to their role in health promotion and disease prevention the safety and low risk toxicological profile of food supplements and functional foods must be ensured. In this context new extraction procedures have been proposed in order to improve the quality and safety of such products as well as to obtain standardized compositions [14]. On the other hand the search for new products to be used as nutraceutical ingredients also underlines the need for new extraction approaches with attention to their environmental impact and consumer safety. In this area our research group recently has explored the use of supercritical CO_2_ for the extraction of natural products such as unsaturated lipids from nuts, and polyphenols from propolis and asparagus [15,16,17,18]. Supercritical carbon dioxide (SCO_2_) could be an alternative and environmentally friendly technique offering several advantages compared to solvent extraction. The process can be performed in closed extractors in the absence of oxygen at controlled temperatures, the CO_2_ can be recycled during the process and allows faster extraction due to its properties of high diffusivity and low density. However, a polar nature of SCO_2_ gives rise to the main limitation of the technique, in that it can extract mainly only lipophilic constituents. On the other hand, this can be at least in part overcome by adding hydrophilic co-solvents that can increase the solubility of polar constituents [19].

In a previous paper we studied the extraction of bioactive constituents from *Eruca sativa* leaves by supercritical CO_2_ technology using different co-solvents [16]. In this paper, with the aim of extracting active compounds from rocket salad and to study the composition of the obtained fractions, we examined the extraction of glucosinolates, phenolics and lipids using a comprehensive approach that employs pretreatment with pure supercritical CO_2_ followed by co-solvent extraction. Water, methanol and ethanol were used at 8% in order to identify the best co-solvent. Analysis by LC-MS allowed the quali/quantitative determination of polyphenol, glucosinolate and lipid constituents extracted under the different conditions. To preliminarily assess the potential use of such extracts for nutraceutical applications the in vitro antioxidant activity was measured using the DPPH assay, while the total reducing capacity was assessed using the FC method.

## 2. Results and Discussion

The constituents of the sample of rocket salad were identified on the basis of HPLC-MS^n^ measurements, comparison with reference compounds and literature data [6,8,9,20]. The identified glucosinolates and phenolics from freeze-dried vegetal material are summarized in Table 1 and Table 2. Comparing the obtained results with the literature, we can observe that the glucosinolate content in our plant material is 19 mg/g and this value is in the range of medium total glucosinolate contents that was reported from 0.25 to 70 mg/g for such plant materials [10]. In our samples the most abundant glucosinolate occurring in leaves were the dimeric form of 4-mercaptobutylglucosinolate (DMB) glucosativin and glucoerucin. Previously published papers indicated that glucoraphanin, glucoerucin and DMB are consistently present in high amounts in the leaves of rocket salad [10,21]. As reported by Bennet et al. [22,23], DMB is considered by some authors an extraction artifact probably formed from 4-mercaptobutylglucosinolate during plant treatment as freeze drying or extraction. The chemical structures of the glucosinolates and flavonols are reported in Table 1 and Table 2 respectively.

**Figure 1:**
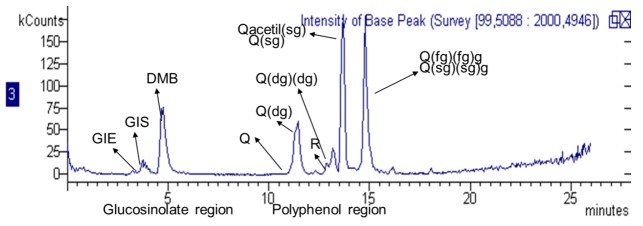
LC/ESI-MS chromatogram of the *Eruca* extract from freeze-dried rocket leaves. Abbreviations of compounds are indicated in Table 1 and Table 2.

A chromatogram representing the main constituents is reported in Figure 1. The method allowed us, due to the MS detection, to indeitify both the glucosinolates without any derivatization, and the flavonoid derivatives. In the proposed method the two groups of constituents elute in different parts of the chromatogram.

### 2.1. Effects of the Co-Solvent on the Glucosinolate Content

**Figure 2:**
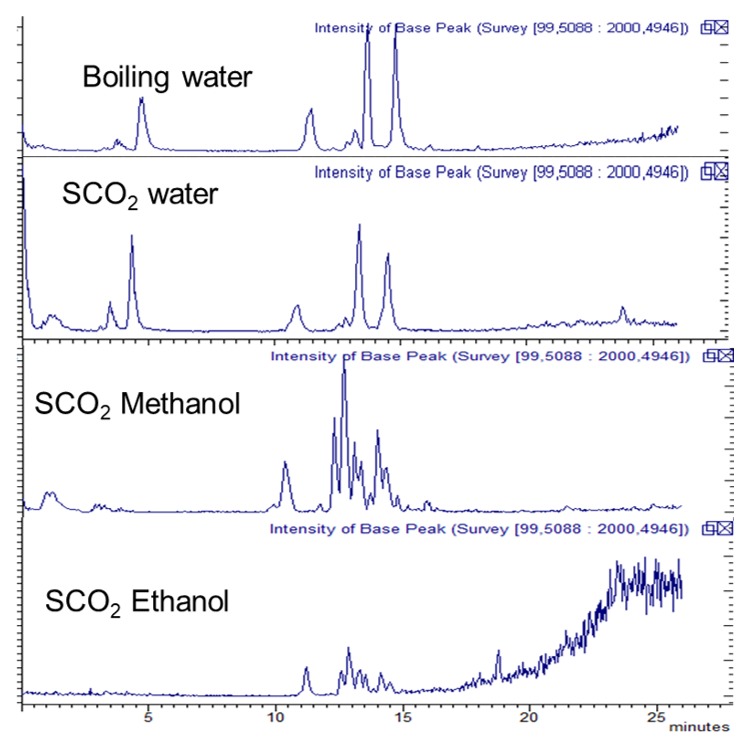
LC/ESI-MS TIC chromatogram of the *Eruca* extract obtained with boiling water SCO_2_ with co-solvent water, methanol and ethanol (8% each).

The extracts obtained using water as co-solvent presented a composition comparable to that obtained in boiling water, while ethanol and methanol were not able to significantly extract glucosinolates. Superimposed chromatograms related to the four type of extraction are reported in Figure 2 showing the differences in composition. As observed, SCO_2_ with alcohols as co-solvent allows minor extraction of the glucosinolates and phenolics from rocket salad leaves as we previously reported. The effect of water as co-solvent and the lower extraction efficiency of alcohols can be explained by the more lipophilic nature of the mixtures of SCO_2_ ethanol and methanol compared to the SCO_2_-water. Furthermore as we can calculate the vapour-liquid equilibria of the CO_2_-solvent system by an equation of state [24] according to literature data [25], which suggests the 8% weight fraction of water is above the solubility of water in CO_2_ at the considered pressure and temperature conditions. For this reason the SCO_2_-water extractions are performed in the presence of an aqueous liquid phase. The same amount (8%) of ethanol or methanol as co-solvents ensure on the other hand the existence of only one phase due to their solubility in CO_2_.

DMB was the most abundant GLS observed in the extracts obtained with water-SCO_2_. Quantitative results of glucosinolate contents in extracts obtained with different co-solvents are reported in Table 3. All the extractions were performed at 45 °C and 30 MPa with respect to the CO_2_ flow rate. Water as co-solvent allowed the extraction of 64% of total glucosinolates in the plant. Methanol and ethanol as co-solvent dramatically decreased the glucosinolates extraction.

Neoglucobrassicin, glucobrassicin and glucocheirolin were not detected in any obtained extract, probably due to their low amount in the freeze-dried starting material. Comparing boiling water with SCO_2_ we observe a relative decrease of the glucosinolate contents and this can be explained by the only partial inactivation of the myrosinase by SCO_2_ as reported for treatment of canola seeds [26], while a complete myrosinase inactivation occurred in boiling water. Thus is possible that partial myrosinase activity was present also during the supercritical fluid extraction explaining the lower extraction yields.

**Figure 3:**
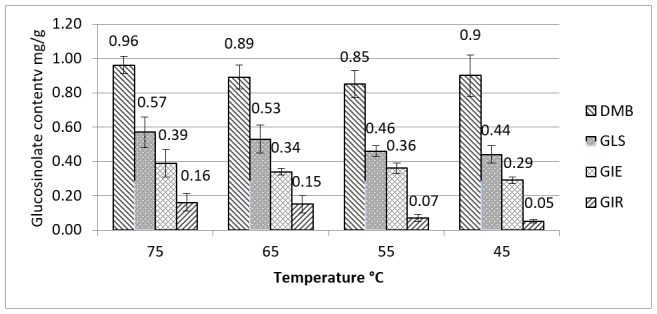
Glucosinolates composition (mg/g) of *Eruca sativa* extract obtained by SCO_2_ at 75 °C, 65 °C and 55 °C, keeping constant the other parameters; differences were not statistically significants (*p* > 0.05). Compounds are abbreviated as follow. Glucoerucin: GlE; Glucorafanin: GlR; Dimeric-4-mercaptobutylglucosinolate: DMB; Glucosativin: GlS.

After the selection of water as co-solvent further parameters were investigated in order to establish conditions affecting the glucosinolate content. The effect of the temperature of supercritical fluid extraction on the glucosinolate composition is shown in Figure 3. The extractions were carried out at 75 °C, 65 °C and 55 °C, keeping constant the other parameters. Pressure was maintained at 30 MPa. No significant differences were observed in the considered temperature range (45–75 °C), thus the extraction using water as co-solvent can be performed at lower temperature with advantages in terms of energy consumption.

**Figure 4:**
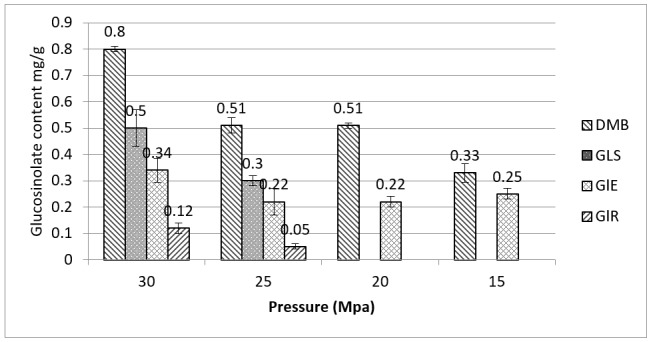
Glucosinolate composition (mg/g) of *Eruca sativa* extract obtained by SCO_2_ at 65 °C and different pressures (*p* < 0.05 for 30 and 25 Mpa contents of DMB, GlS, GlE and GIR). Compounds are abbreviated as follow. Glucoerucin: GlE; Glucorafanin: GlR; Dimeric-4-mercaptobutylglucosinolate: DMB; Glucosativin: GlS.

The effect of pressure on the glucosinolate extraction was studied and results are reported in Figure 4, (the temperature was 65 °C). As we expected, at the higher pressure there is a significant increase in the glucosinolate content, due to the higher solubility of such hydrophilic constituents in the supercritical-cosolvent mixture.

The results obtained in the glucosinolate extraction showed that use of supercritical CO_2_ with water as co-solvent can be useful for the production of extracts for nutraceutical purposes. The conventional procedures use solvents and high temperature to inactivate myrosinase. The new approach allows one to work at lower temperatures and milder pressure and can be a starting point to also develop an extraction for industrial applications.

### 2.2. Effect of the Extraction Conditions on the Phenolic Content

The effect of temperature of the supercritical fluid extraction on the phenolic composition is shown in Table 4. The compositions of the extracts at 75 °C, 65 °C, 55 °C and 45 °C (keeping pressure at 30 MPa) are reported. Compared with ultrasound-assisted extraction (UAE) the extraction of phenolics using methanol-water is in general less efficient, being 57% of the total polyphenols of the plant. Some differences can be observed taking considering single compounds. Leucodelphynidine is extracted in similar amounts at 65 and 75 °C, while a significant decrease in the extraction is observed at lower temperature, being the 50% less compared to methanol-water UAE. Quercetin-3-(6-sinapoyl glucoside) is extracted in comparable amounts only at 75 °C, while at lower temperature the extraction yield is less than 50% compared to methanol-water UAE. Quercetin is extracted with very good yield under all conditions, with 70% of yield at 45 and 55 °C. Similar behavior is also observed for kaempferol and isorhamnetin with less extraction efficiency at lower temperature.

The effect of pressure on phenolic extraction was studied and results are reported in Table 5 (at a constant temperature of 65 °C, 8% water as co-solvent). Non-significant differences were observed in the range of 15 to 30 Mpa.

The more complex glicosylated derivatives such a as rutin, Q(dg)(sg) were less efficiently extracted probably due to their high polar nature.

### 2.3. Polyunsaturated Lipid Derivatives of the SCO_2_ Extracts

**Figure 5:**
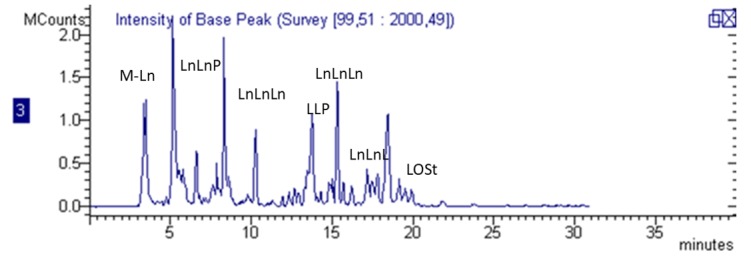
LC/APCI-MS TIC of the SCO_2_ extract from *Eruca* leaves.

Quali/quantitative analysis of unsaturated lipids was performed on the obtained SCO_2_ extracts. Monoacylglycerols and tryglycerides were tentatively identified on the basis of their APCI-MS spectra and comparison with literature data [27,28,29]. The main constituents were monoacylglycerols of linolenic acid, and triacylglycerols containing at least one linolenic or linoleic acid residue. The fatty acid composition of the three extracts were qualitatively similar, being characterized by a higher percentage of linoleic (L), linolenic (Ln), oleic (O), palmitic (P), stearic (S) and steridonic (St) acid. The samples extracted by pure SCO_2_ and with ethanol and methanol as co-solvent have high contents in unsaturated fatty acids. However, the samples extracted using water as co-solvent have only traces of lipids (data not shown). The average mg/g values of the identified constituents in the extracts are reported in Table 6 and a typical chromatogram of an extract is reported in Figure 5.

### 2.4. Antioxidant Activity of the Obtained Extracts

In vitro antioxidant activity was measured using the DPPH test, and results are reported in Table 7 and Table 8.

Similar antioxidant activity was measured for the extracts obtained at the same pressure and temperature conditions (30 MPa and 45 °C) with or without co-solvents (Table 7). The antioxidant activity can be ascribed to unsaturated lipid constituents for the most lipophilic extracts while for water can be ascribed to polyphenols and glucosinolates. Considering the SCO_2_-water extract, it presents higher levels of glucosinolates as well as leucodelphynidine and quercetin that are known for their antioxidant effects. In general the obtained data show a correlation considering the phenolic and glucosinolate contents and DPPH and FC test effects (Table 8).

## 3. Materials and Methods

### 3.1. Raw Material

Rocket salad leaves were supplied by the agri-company “La Marostegana”, located in Piazzola di Brenta (Italy). Raw material was crushed with a kitchen grinder and quickly frozen at −25 °C. Material was then lyophilized and milled with a mortar. After extraction, the samples were kept in a closed storage container at 4 °C for further analysis.

### 3.2. Chemicals

CO_2_ (purity 99.99%) used as supercritical solvent was purchased from Rivoira (Milano, Italy). Ethanol, methanol, acetonitrile, formic acid, and ethanol were provided by Carlo Erba (Milano, Italy), WWR, BDH Prolabo solvents (Milan, Italy), and J.T. Baker Fisher Scientific (Milano, Italy). Water used as co-solvent was Milli-Q quality. 1,1-Diphenyl-2-picrylhydrazyl (DPPH), rutin, chlorogenic acid were obtained from Sigma Aldrich (Milano, Italy). Glucoerucin, glucosativin, and gluconapin were obtained from Phytolab GMB (Vestenbergsgreuth, Germany).

### 3.3. Supercritical Fluid Extraction with Different Co-Solvents

The extraction procedures involved several steps and was performed in a self built extractor assembled by the Industrial Engineering group of the University of Padova as detailed in previous publications [17,19]. First, the stainless steel extraction cell (16) was filled with 0.5 ± 0.05 g of lyophilized rocket salad powder. Then, CO_2_ was pumped by a high pressure pump (8) at a constant CO_2_ flow rate of 0.3 ± 0.05 kg h^−1^. Pressure was controlled by two gauges (6, 14). A thermo-resistance placed around the extraction cell maintained the desired temperature, which was measured in the internal flow before and after the cell (15, 17). Once the experimental conditions were reached, a pre-treatment consisting in a pure supercritical CO_2_ extraction during 15 min was carried out, in order to extract the low polarity CO_2_-soluble compounds. A batch of vegetal material was used for the pretreatment with CO_2_ then the pre-extracted material was used for further extraction. On the part of the vegetal material, the co-solvent, in a proportion of 8% with respect to the CO_2_ flow rate, was pumped by an intelligent pump (13) and mixed with CO_2_ before the extraction cell. Thus three different extractions were performed after pre-treatment of plant material using water, ethanol and methanol at 8% as co-solvents. After extraction, the mixture of CO_2_, co-solvent and extract was expanded by a valve inserted in a water bath at 40 °C, avoiding CO_2_ freezing caused by sudden pressure reduction (18). The extract and the co-solvent were collected (20) in 12 mL of a solvent (the same used as a co-solvent). CO_2_ gas at atmospheric pressure passed through a flow meter (21) before being vented. The extract was filtered through a 0.20 µm filter (Ministart, Milan, Italy) and the co-solvent was evaporated by a rotary evaporator. Three different extracts were obtained at 65 °C and 30 MPa. Then on other pretreated material extraction with water as co-solvents were performed changing pressure and temperature conditions, namely 55, 65 and 75 °C at constant pressure of 30 MPa, and keeping a constant temperature at 65 °C with pressures of 15, 20, 25 and 30 MPa.

### 3.4. Quantification of the Phenolic, Glucosinolate and Lipids Contents

The extraction protocol that was used for comparison purposes for the analysis of phenolic compounds was based on previous papers [30,31,32] and consists of 20 min sonication of the freeze-dried leaves (0.100 ± 0.001 g) in an ultrasound system with 10 mL of 70% methanol. The extraction was repeated twice. After centrifugation, the supernatant liquids were collected and volume was adjusted to 25 mL in a volumetric flask. The final water content of the solution was adjusted to 50% to increase chromatographic peak resolution. Solutions were filtered through 0.45 μm and used for the HPLC analysis. For the quantification of the glucosinolates content, the freeze-dried vegetal material (0.100 ± 0.005) was added to internal standard (gluconapin solution 100 μg/mL) and extracted in boiling water (10 mL) during 10 min controlling the temperature at 100 °C in order to inactivate enzymatic activities (myrosinase) Extraction was repeated twice. The supernatant was removed after centrifugation and volume was adjusted to 25 mL.

Methanol was the solvent used for lipids extraction from the rocket leaves. 0.050 ± 0.001 g of the freeze-dried vegetable material was sonicated with 2 mL of methanol in a flask, the extraction was repeated twice. The sample was centrifuged and the supernatant was analysed by HPLC-MS.

To carry out the quali/quantitative analysis of glucosinolates, phenols and lipids in the extracts, different HPLC-MS methods were used. The measurements were performed on a Varian 212 series chromatograph (Varian inc, Palo Alto, CA, USA) equipped with Prostar 430 autosampler and a MS-500 mass spectrometer with an ion trap analyser. MS spectra were recorded in negative and positive ion mode in the *m*/*z* range 50–2000. Fragmentation of the main ionic species were obtained by the turbo data depending scanning (TDDS) function. The ESI ion source was used for phenolic and glucosinolates analysis. An Eclipse Plus C-18 (2.1 × 150 mm) 3.5 μm column (Agilent, Santa Clara, CA, USA) was used as stationary phase. For glucosinolates and phenolics mobile phase were solvent A (water 0.1% formic acid) and solvent B (Acetonitrile). The elution gradient started at 90% A then decreased to 0% over 25 min. Quantification of phenolic constituents was obtained with the method of calibration curve: rutin was used as external standard in the range 0.5–10 μg/mL at five different concentration. Its calibration curve was Y = 134292X + 512 (R^2^ = 0.9998). For the quantification of glucosinolates we used the method of internal standard: gluconapin was used as internal standard and the calibration curve was obtained preparing solutions with different ratio of gluconapin (IS) and each different glucosinolate from standard solution at 1.04 mg/mL. Calibration curve (area ratio Y vs amount fration X) was Y = 6.169X + 0.6691 (R^2^ = 0.9985).

The APCI was used for lipid analysis. Mobile phase were A (acetonitrile) and B (isopropanol) and the gradient elution profile was as follows: 0 min, 100% A; 10 min, 40% A; 20 min, 40% A; 22 min, 15% A; 30 min, 15% A. To obtain semiquantitative data as reference compound caprylic diglyceride was used and the calibration curve was obtained in the 3–173 μg/mL range (Y = 8 × 10^−6^X + 16; R^2^ = 0.9998).

### 3.5. DPPH Assay

The scavenging activity towards the 1,1-diphenyl-2-picrylhydrazyl radical was measured by modifying a previously used protocol [32]. Rutin was used as standard reference in order to compare obtained results with a known antioxidant compound. A linear range of concentration vs. % decrease of absorbance was observed and was used for the determination using rutin solutions in the range 0.26–52 μg/mL. Methanol solution of *Eruca sativa* extracts were used for the assay and the relative % decrease of absorbance was calculated. The activity was expressed as mg/g equivalent of rutin.

### 3.6. Folin-Ciocalteau

Total phenolic content and other oxidation substrates were examined using the Folin Ciocalteau (FC) reagent. We used a previously described method [32]. The FC assay relies on the transfer of electrons in alkaline medium from phenolic compounds to phosphomolybdic/phosphotungstic acid complexes, which are determined at 765 nm. Antioxidant reducing capacity is expressed as gallic acid equivalents (GAE).

## 4. Conclusions

Glucosinolate-containing vegetables are highly considered due to the health promoting effects of their constituents. Due to the presence of glucosinolates, polyphenols and unsaturated fatty acids *Eruca sativa* can be considered a good starting material for nutraceutical production. Up to now the papers related to the extraction procedures from this species or other glucosinolate-containing vegetables are mainly related to analytical approaches, and only limited literature is related to the extraction procedures that can be useful for production of extracts for use in nutraceutical, cosmetic or pharmaceutical applications. For this reason the data of the present study, that correlates an innovative extraction procedure with the phytochemical composition of the obtained extracts, demonstrates the good opportunity offered by the supercritical CO_2_ extraction with co-solvents as a technique for preparing nutraceutical ingredients. The search for new health-promoting extracts to be used in food and nutraceutical products is closely connected with the development of new extraction techniques that can reduce the use of solvents thus increasing the safety of the final products. Supercritical fluid extraction is a very promising approach in this area and can be useful, expecially for the extraction of the most lipophilic natural products also from agro food chain waste [33,34,35].

The limitations of the extraction capacity of supercritical CO_2_ can be at least in part overcome using co-solvents and we used this approach for extracting the rocket salad leaves. It was found that phytoconstituents are extracted in sufficient amount compared with traditional techniques but avoiding the use of solvents. The LC-MS analysis allowed the determination of different glucosinolates and revealed that 64% of the glucosinolates of the plant are extracted compared to boiling water, probably due to only a partial inactivation of the enzyme myrosinase. A 54% of the flavonoid constituents are extracted compared with methanol/water UAE extraction. Only the more lipophilic flavonoids like leucodelphinidin, quercetin, kaempferol, and isorhamnetin are extracted in the amount comparable with the methanol-water UAE extraction.

The extraction with SC-CO_2_ and water allows one to work at lower temperatures than conventional procedures. Under the experimental conditions used in this work we observed no differences in the extracted amounts of glucosinolate and phenolics when the temperature ranged from 45° to 75 °C at 30 with a constant pressure of 30 MPa. Otherwise, by increasing the pressure from 15 to 30 MPa a higher amount of glucosinolates can be extracted, but not flavonoids. Significant antioxidant effects wer observed in the in vitro DPPH test and the results directly correlated with the polyphenol and glucosinolate extraction. Finally, on the basis of the results, this extraction procedure involving different steps can be suitable to obtain extracts with a complete range of-health promoting constituents, namely lipids, polyphenols and glucosinolates, with a green, food compatible process. Further application of this innovative extraction protocol will be done in order to study the opportunity to apply it to different plant sources containing glucosinolates.

## Figures and Tables

**Table 1:** Glucosinolates identified by LC/ESI-MS^n^ in freeze-dried rocket leaves.

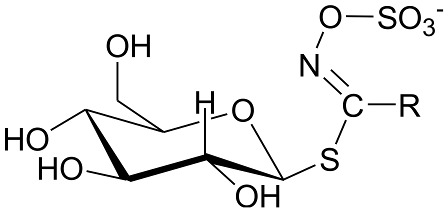				
Glucosinolate	Abbreviation	[M − H]^−^	mg/g	R
Glucoerucin	GlE	420	0.68 ± 0.01	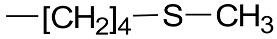
Glucorafanin	GlR	436	0.12 ± 0.02	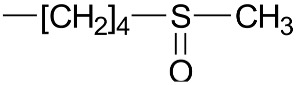
Dimeric 4-mercaptobutylglucosinolate	DMB	811	16.46 ± 0.99	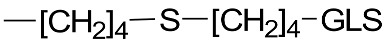
Glucocheirolin	GlChe	438	0.09 ± 0.01	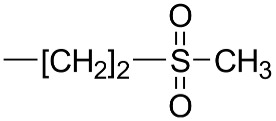
Glucosativin	GlS	406	1.05 ± 0.02	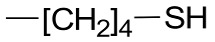
Neoglucobrassicin	NeoGlB	477	0.11 ± 0.01	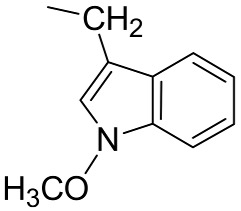
Glucobrassicin	GlB	447	0.16 ± 0.01	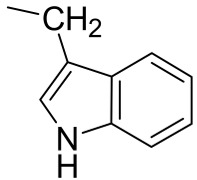

**Table 2:** Phenolics identified by LC/ESI-MS^n^ in freeze-dried rocket leaves. ORut = *O*-rutinoside.

Flavonols Structure										
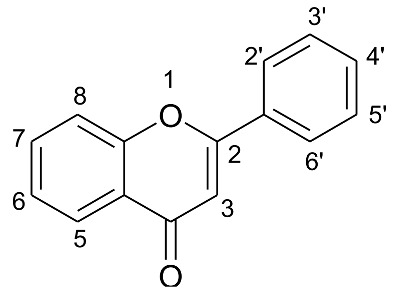	Position		3	5		7	3′		4′	5′
	Quercetin		-OH	-OH		-OH	-OH		-OH	-
	Rutin		-ORut	-OH		-OH	-OH		-OH	-
	Keampferol		-OH	-OH		-OH	-		-OH	-
	Isorhamnetin		-OH	-OH		-OH	-		-OH	-OCH_3_
**Phenolics**		**Abbreviation**			**[M-H]^−^**			**mg/g**		
Quercetin-3-(2-feruloyl-glucoside)-3′-(6-feruloylglucoside)-4′glucoside		Q(fg)(fg)g			1139			0.09 ± 0.00		
Quercetin-3,4′-diglucoside-3′ (6-sinapoyl-glucoside)		Q(dg)(sg)			993			0.39 ± 0.02		
Rutin		R			609			0.12 ± 0.01		
Luecodelphynidin		L			321			0.49 ± 0.01		
Quercetin-3-(6-acetylsinapoyl-diglucoside)		Qacetil(sg)			831			0.27 ± 0.02		
Quercetin(sinapoyl-glucoside)(sinapoyl-glucoside)-glucoside		Q(sg)(sg)g			1199			0.59 ± 0.03		
Quercetin-3-(6-sinapoyl-glucoside)		Q(sg)			669			0.26 ± 0.02		
Quercetin		Q			301			0.29 ± 0.02		
Kaempferol		K			285			0.19 ± 0.01		
Isorhamnetin		IsoR			315			0.19 ± 0.02		
Quercetin-3,4′-diglucoside		Q(dg)			625			0.32 ± 0.01		

**Table 3:** Glucosinolates composition (mg/g) of *Eruca sativa* extract obtained by SCO_2_ with different co-solvents.

	Glucosinolate Content mg/g			
	Boiling Water	SCO_2_ Water	SCO_2_ Methanol	SCO_2_ Ethanol
GlE	0.68 ± 0.01 ^a^	0.29 ± 0.01 ^b^	n.d.	n.d.
GlR	0.12 ± 0.02 ^a^	0.05 ± 0.02 ^b^	n.d.	n.d.
DMB	16.46 ± 0.99 ^a^	11.00 ± 0.01 ^b^	0.30 ± 0.01 ^c^	n.d.
GlS	1.05 ± 0.02 ^a^	0.44 ± 0.02 ^b^	n.d.	n.d.

Compounds are abbreviated as follow. Glucoerucin: GlE; Glucorafanin: GlR; Dimeric-4-mercaptobutylglucosinolate: DMB; Glucosativin: GlS. Different letters indicate significant differences (*p* > 0.05); n.d.: not detected.

**Table 4:** Phenol composition (mg/g) of *Eruca sativa* extract obtained by SCO_2_ at different temperatures.

Temperatures	SCO_2_ 8% Water 75 °C	SCO_2_ 8% Water 65 °C	SCO_2_ 8% Water 55 °C	SCO_2_ 8% Water 45 °C	Methanol Water UAE
Q(fg)(fg)g	0.07 ± 0.02	0.07 ± 0.01	0.07 ± 0.01	0.07 ± 0.01	0.09 ± 0.00
Q(dg)(sg)	0.13 ± 0.01 ^a^	0.15 ± 0.02 ^a^	0.13 ± 0.01 ^a^	0.11 ± 0.01 ^a^	0.39 ± 0.02 ^b^
R	0.06 ± 0.01 ^a^	0.03 ± 0.01 ^b^	0.03 ± 0.01 ^b^	0.06 ± 0.01 ^a^	0.12 ± 0.01 ^c^
L	0.42 ± 0.03 ^a^	0.44 ± 0.04 ^a^	0.28 ± 0.03 ^b^	0.26 ± 0.01 ^b^	0.49 ± 0.02 ^a^
Qacetil(sg)	0.12 ± 0.01 ^a^	0.07 ± 0.01 ^b^	0.07 ± 0.05 ^b^	0.07 ± 0.01 ^b^	0.27 ± 0.02 ^c^
Q(sg)(sg)g	0.10 ± 0.01 ^a^	0.08 ± 0.01 ^a^	0.09 ± 0.04 ^a^	0.07 ± 0.01 ^a^	0.59 ± 0.03 ^b^
Q(sg)	0.21 ± 0.02 ^a^	0.12 ± 0.01 ^b^	0.11 ± 0.01 ^b^	0.11 ± 0.01 ^b^	0.26 ± 0.02 ^c^
Q	0.28 ± 0.02 ^a^	0.27 ± 0.05 ^a^	0.20 ± 0.02 ^b^	0.21 ± 0.01 ^b^	0.29 ± 0.02 ^a^
K	0.18 ± 0.01 ^a^	0.18 ± 0.02 ^a^	0.14 ± 0.03 ^b^	0.11 ± 0.03 ^b^	0.19 ± 0.01 ^a^
IsoR	0.19 ± 0.01 ^a^	0.19 ± 0.01 ^a^	0.16 ± 0.03 ^a^	0.14 ± 0.03 ^b^	0.19 ± 0.02 ^a^
Q(dg)	0.07 ± 0.01 ^a^	0.09 ± 0.01 ^a^	0.04± 0.01 ^a^	0.04 ± 0.01 ^a^	0.32 ± 0.01 ^b^

Different letters indicate when differences were statistically significant (*p* < 0.05) between different extraction temperatures and UEA extraction. Compounds are indicated as follows. Quercetin-3-(2-feruloyl-glucoside)-3′-(6-feruloylglucoside)-4′glucoside, Q(fg)(fg)g; Quercetin-3,4′-diglucoside-3′(6-sinapoyl-glucoside): Q(dg)(sg); Rutin:R; Luecodelphynidin: L; Quercetin-3-(6-acetylsinapoyl-diglucoside): Qacetil(sg). Quercetin(sinapoyl-glucoside)(sinapoyl-glucoside)-glucoside: Q(sg)(sg)g; Quercetin-3-(6-sinapoyl-glucoside):Q(sg); Quercetin: Q; Kaempferol: K; Isohramnetin: IsoR; Quercetin-3,4′-diglucoside:Q(dg).

**Table 5:** Phenol composition (mg/g) of *Eruca sativa* extract obtained by 8% water SCO_2_ at different pressures.

Pressure (MPa)	30	25	20	15
Q(fg)(fg)g	0.07 ± 0.01	0.07 ± 0.01	0.07 ± 0.01	0.07 ± 0.01
Q(dg)(sg)	0.15 ± 0.01	0.13 ± 0.02	0.13 ± 0.03	0.13 ± 0.04
R	0.09 ± 0.01	0.07 ± 0.01	0.08 ± 0.01	0.07 ± 0.01
L	0.44 ± 0.04 ^a^	0.41 ± 0.03 ^a^	0.39 ± 0.04 ^a^	0.29 ± 0.03 ^b^
Qacetil(sg)	0.09 ± 0.01	0.09 ± 0.02	0.07 ± 0.01	0.08 ± 0.01
Q(sg)(sg)g	0.08 ± 0.01	0.08 ± 0.01	0.08 ± 0.01	0.10 ± 0.02
Q(sg)	0.17 ± 0.01	0.14 ± 0.01	0.11 ± 0.01	0.11 ± 0.02
Q	0.23 ± 0.03	0.22 ± 0.02	0.22 ± 0.03	0.19 ± 0.03
K	0.18 ± 0.01	0.17 ± 0.01	0.16 ± 0.01	0.16 ± 0.01
IsoR	0.18 ± 0.01 ^a^	0.10 ± 0.01 ^b^	0.09 ± 0.01 ^b^	0.11 ± 0.02 ^a^
Q(dg)	0.09 ± 0.01	0.07 ± 0.01	0.06 ± 0.01	0.06 ± 0.01

Different letters indicate significant differences (*p* > 0.05). Compounds are indicated as follows. Quercetin-3-(2-feruloyl-glucoside)-3′-(6-feruloylglucoside)-4′glucoside, Q(fg)(fg)g; Quercetin-3,4′-diglucoside-3′(6-sinapoyl-glucoside): Q(dg)(sg); Rutin:R; Luecodelphynidin: L; Quercetin-3-(6-acetylsinapoyl-diglucoside): Qacetil(sg). Quercetin(sinapoyl-glucoside)(sinapoyl-glucoside)-glucoside: Q(sg)(sg)g; Quercetin-3-(6-sinapoyl-glucoside):Q(sg); Quercetin: Q; Kaempferol: K; Isohramnetin: IsoR; Quercetin-3,4′-diglucoside:Q(dg).

**Table 6:** Lipids composition (mg/g) of the extracts obtained by SCO_2_ with methanol or ethanol as co-solvents.

Lipid Derivative	SCO_2_	SCO_2_-Ethanol	SCO_2_-Methanol
**Monoacylglycerol of Ln**	0.73 ± 0.01 ^a^	2.04 ± 0.01 ^b^	1.51 ± 0.02 ^c^
**LnLnP**	0.59 ± 0.02 ^a^	1.86 ± 0.05 ^b^	1.50 ± 0.01 ^b^
**LLnP**	0.58 ± 0.01 ^a^	1.99 ± 0.03 ^b^	1.49 ± 0.02 ^b^
**LLP**	0.60 ± 0.01 ^a^	1.81 ± 0.02 ^b^	1.50 ± 0.01 ^b^
**LnLnLn**	1.21 ± 0.06 ^a^	6.32 ± 0.09 ^b^	1.77 ± 0.03 ^c^
**LnLnL**	0.78 ± 0.01 ^a^	2.47 ± 0.06 ^b^	1.55 ± 0.02 ^c^
**LOSt**	0.57 ± 0.01 ^a^	1.88 ± 0.09 ^b^	1.52 ± 0.01 ^c^

Abbreviations indicate linoleic (L), linolenic (Ln), oleic (O), palmitic (P), stearic (S) and steridonic (St) acid. Different letters indicate significant differences (*p* > 0.05).

**Table 7:** DPPH test results compared with amount of *Eruca sativa* constituents in extracts obtained with SCO_2_ at 30 MPa and 45 °C using different co-solvents.

Extraction System	DPPH (Rutin Equivalent mg/100 g of Extract)	Gallic Acid Equivalent (mg/100 g)	Glucosinolate Content (mg/100 g)	Polyphenols Content (mg/100 g)	Lipids Content (mg/g)
**SCO_2_-Water**	29.5 ± 1	659 ± 3	178 ± 3	61 ± 2	n.d.
**SCO_2_-Methanol**	33.4 ± 2	122 ± 1	30.0 ± 1	n.d.	1084 ± 80
**SCO_2_-Ethanol**	33.0 ± 2	82 ± 2	n.d.	n.d.	1840 ± 50
**SCO_2_**	13.0 ± 2	76 ± 1	n.d.	n.d.	448 ± 10

n.d. Not determined.

**Table 8:** Antioxidant activity and total secondary metabolite contents in *Eruca sativa* extract obtained by SCO_2_-water at different pressures and a constant temperature of 65 °C.

Pressure (MPa)	30	25	20	15
DPPH (rutin equivalent mg/100g)	82 ± 2	54 ± 3	58 ± 4	55 ± 3
FC Gallic acid equivalent (mg/100g)	659 ± 3	644 ± 6	452 ± 1	378 ± 2
Phenolics (HPLC) (mg/100g)	1460 ± 12	1230 ± 11	1200 ± 1	1160 ± 13
Glucosinolates (HPLC) (mg/100g)	1770 ± 6	1308 ± 3	730 ± 1	740 ± 1.2
